# Mental health impact of COVID-19 on Saudi families and children with special educational needs and disabilities in Saudi Arabia: A national perspective

**DOI:** 10.3389/fpubh.2022.992658

**Published:** 2022-09-27

**Authors:** Shuliweeh Alenezi, Mohamad-Hani Temsah, Ahmed S. Alyahya, Ahmad H. Almadani, Afnan Almarshedi, Maha S. Algazlan, Faisal Alnemary, Fahad A. Bashiri, Samah Hazem Alkhawashki, Maram Hani Altuwariqi, Rafif Alsedrani, Aqeel Alkhiri, Mohammed Alarabi

**Affiliations:** ^1^Department of Psychiatry, College of Medicine, King Saud University, Riyadh, Saudi Arabia; ^2^Department of Psychiatry, King Saud University Medical City, King Saud University, Riyadh, Saudi Arabia; ^3^SABIC Psychological Health Research and Applications Chair, Department of Psychiatry, College of Medicine, King Saud University, Riyadh, Saudi Arabia; ^4^Pediatric Department, King Saud University Medical City, King Saud University, Riyadh, Saudi Arabia; ^5^Department of Psychiatry, Eradah Complex for Mental Health, Riyadh, Saudi Arabia; ^6^Psychiatry Department, Prince Sultan Military Medical City, Riyadh, Saudi Arabia; ^7^Autism Center of Excellence, Riyadh, Saudi Arabia; ^8^Pediatric Neurology Division, Department of Pediatrics, College of Medicine, King Saud University, Riyadh, Saudi Arabia; ^9^Department of Mental Health, Al Qunfudah General Hospital, Al Qunfudah, Saudi Arabia

**Keywords:** COVID-19, children with special educational needs and disabilities, mental health, Saudi Arabia, coping behavior

## Abstract

**Introduction:**

The COVID-19 pandemic revealed a multidimensional impact on mental health due to health concerns, social distancing and lockdowns, job loss, and limits in institutional support. Accordingly, COVID-19 may disproportionally impact families with special educational needs and disabilities (SEND) due to the already high prevalence of mental health conditions in children with SEND and their parents. Hence, it is essential to determine the short-term impact of the pandemic on the mental health of families with SEND to identify their ongoing health, including psychological wellbeing and support needs. The current study examines the anxiety level and concerns of children with SEND and their parents living in Saudi Arabia.

**Methodology:**

A cross-sectional national study design was utilized as a part of an international consortium using an online Arabic survey. Data were collected from the Ministry of Human Resources and Social Development beneficiaries from May to July 2020. The sample consisted of 1,848 parents of children with SEND aged between 1 and 18 years (mean = 9.66; SD = 4.31). A descriptive and bivariant analysis is reported.

**Results:**

Parental worries on all those concerns when the pandemic started were significantly higher than before the pandemic, *p* < 0.050. Parental-perceived general anxiety had risen significantly across time, *p* < 0.001, and their perceived anxiety when the pandemic started exceeded their anxiety before the pandemic, *p* < 0.001. The general anxiety of children with SEND had risen significantly across time (from before the pandemic to when it had started to during the pandemic), *p* < 0.001. The children's general worries at the start of the pandemic had correlated significantly and positively with their anxiety, adaptive, maladaptive, and coping efficacies, and parental anxiety scores, *p* < 0.010 each.

**Conclusion:**

Anxiety levels were high in SEND and their caregivers before and during COVID-19. At the start of the pandemic, the anxiety, adaptive, maladaptive, coping efficacies, and parental anxiety scores of children with SEND were significantly and favorably correlated. These findings support the notion of SEND-specific anxiety and patterns of coping in SEND and their caregivers. The notion also attests to the institutional support required for this specifically vulnerable population during epidemics.

## Introduction

The SARS-CoV-2 virus (COVID-19) has had a catastrophic impact lasting worldwide since it was first reported in China in December 2019 (1). The WHO recognized the potential for human-to-human transmission of the virus on January 14, 2020 (2), and later confirmed this mode of transmission on January 22, 2020 (3). Few months later, the WHO declared COVID-19 a global pandemic and urged countries to take needed precautions to contain its spread (4).

In Saudi, the first case was reported on March 2, 2020; the pandemic rapidly evolved despite methods taken to contain it. By the time of authoring this article, there were 546,612 reported cases in the country. The government implemented several preventive and restrictive measures to slow down the spread, including but not limited to wearing face masks, social distancing, travel, and movement restrictions, withholding social events and large gatherings, closure of schools and universities, and imposing partial curfews at varied times. A partial curfew was first implemented on March 23, 2020, which was 12 h a day; this was later extended to be a 24-h curfew on April 6th for three consecutive weeks (5, 6).

Despite the initial support from parents in the previous measures, demands associated with caregiving increased dramatically as the pandemic evolved. Caregivers were left facing challenges in balancing work, childcare, and home-schooling without the support of grandparents, extended family, and teachers. Home-schooling had added to the psychological distress of parents who had to balance schooling along with their work and social commitment compared to those who did not have to home-school their children (7).

The challenges of families with children of special educational needs and disabilities (SEND) are more pronounced and have been going on for an extended period prior to the pandemic and its associated restrictive measures, which only made things harder. SEND had been dealing with prevailing challenges, including the accessibility of essential services involving healthcare, transportation, communication, accommodation, respite care, community support, and mobility in the public domain (8–12). They are a particularly vulnerable population due to their greater health needs and their dependency on community-based services; taking virtual services and telemedicine as an example which have been the main method of communication amid the pandemic, children with impairments in communication, attention, and/or coordination, required additional specific accommodations to utilize such services. This, in turn, contributed to the worsening of their mental health status, thus, increasing the psychological burden on caregivers (13). In consideration, the WHO issued a briefing emphasizing the need to include measures to ensure the inclusion of individuals with special needs in the pandemic response. The briefing described four areas of action: (1) mainstreaming of the disability in all COVID responses and recovery, together with targeted actions; (2) ensuring accessibility of information, facilities, services, and programs; (3) ensuring meaningful consultation with the active participation of persons with disabilities; and (4) establishing accountability mechanisms to ensure disability inclusion in all stages of the response and recovery process (14). Additional negative sequels of the pandemic on individuals with disabilities have been identified which included weight loss, muscle weakness, and/or increased tone, low mood, learning and social regression, less fitness, and poor behavior (15).

Previous studies on health-related disasters had shown that the disease-acquiring responses, such as quarantine and self-isolation, could be significantly traumatizing to children and their caregivers. Changes in the daily routine and changes in the access to health and social-related services can pose significant distress to a proportion of children with neurodevelopmental disabilities (NDDs_; the changes can include but are not limited to a reduction in the availability of formal and informal supports, including contact with close family members (16). An Australian study evaluated the impact of the pandemic on parents of children with NDD; the results showed that 76.9% of parents reported that the health and wellbeing of their children were negatively impacted by COVID-19, with 18.8% reporting a need to increase the dosage of medication administered to their children. Additionally, 76.1% of parents reported impacts on their own mental health (17).

Adverse effects among caregivers of children with special needs, medical complexity, and mental health challenges have also been noted, including an increased prevalence of depression, anxiety, and stress syndromes (18). A recent study examined caregiver's strain before and amid the pandemic and reported a significantly higher prevalence of depressive symptoms among 62.5% of caregivers whereas 20.5 and 36.4% were indicated for anxiety and stress symptoms, respectively (19), in contrast to 37.1 and 20.1%, reported, respectively, by two studies conducted during the initial period of the lockdown among the general population (20, 21). It was also noted that an extended period of the outbreak was not associated with a change in depression scores among the general population (22).

Overall, the COVID-19 pandemic continues to negatively impact the psychological health of family caregivers in general and caregivers of children with special needs; hence, this study aims to assess the impact of the COVID-19 pandemic on the mental health of individuals with disability and their caregivers by assessing the worries of parents and the children of SEND and the coping mechanisms implemented by children with SEND.

## Methodology

### Study settings

The current study is a part of an international collaboration in response to COVID-19 pandemic and its impact on children with disabilities. More than 30 countries participated in the study, and 60 researchers worldwide participated (www.specialneedscovid.org). An international ethics approval was obtained for a larger study from the UniDistance Suisse, Switzerland, titled, “How Families with Children with Special Needs are coping with the COVID-19 Pandemic: An International Online Study.” The main project was initially designed by Van Herwegen et al. (Principal Investigators) in English, German, and French (23). The principal investigators invited international researchers from 25 countries to collaborate on the study, resulting in translation into 15 additional languages, including Arabic and Spanish. The entire survey can be accessed on the OSF website (https://osf.io/5nkq9/).

### Research design

This is a secondary analysis of a cross-sectional study design that was utilized as a part of an international consortium using an Arabic survey. A descriptive and bivariate analysis is reported. This is the first published report from this data set.

### Data collection

Data collection occurred from May 2020 to July 2020. The survey took about 35 min to complete. Parents were requested to enter an identifying code at the end of the survey (initials of their name and date of birth). If participants desired, they could withdraw their anonymous data at any time after completing the survey using this code. The identifying numbers were checked to verify that no parent completed the survey more than once. The survey also included an additional attention check question to guarantee that the participant replies were valid. Participants who did not pass the attention test were eliminated from the study.

### Population and sampling methods

The population of the study was drawn from caregivers of SEND in Saudi Arabia. The survey was sent to SEND caregivers *via* text messages to all beneficiaries of the Ministry of Human Resources and Social Affairs, the social media accounts of the Autism Center of Excellence, and the Authority for Persons with Disabilities.

### Instrument (reliability and validity)

The study was conducted through an online survey (in Arabic) that was spread to caregivers of SEND. Cronbach's alpha test was used to assess the internal consistency of the measured questionnaires, and the exploratory factor analysis (EFA) was applied to these measured psychometric measures to assess their validity and factorial structure as well as their unidimensionality.

### Overview of the different sections of the survey

There were 111 open-ended and closed-ended questions in the survey. Only those queries that are pertinent to the current investigation were described. The survey was subdivided into the following three main sections:

- Section A: Respondents were asked about the background of their children with SEND, including demographic data (such as gender, age, parental education, work status, and whether they lived in an urban or rural area), verbal ability, and the medical history of the children with SEND (e.g., medical diagnoses and other health issues).
- Section B: Questions were included about the anxieties of the participating parents and their children with SEND. Parents were asked to determine on a 5-point Likert scale (with 1 = no anxiety to 5 = very anxious).
- Section C: Questions were focused on the activity and response of the children with SEND during the COVID-19 period (social distancing, ability to cope with shifts in routine, being bored, the possibility of a parent getting sick, and fears of losing of institutional services). In this section, participants were requested to submit a response for three different time points: before the pandemic, at the start of COVID-19, and at the time of the survey.

## Analysis

Data analysis was conducted with the statistical package for the social sciences (SPSS), utilizing a significant value of *p* ≤ 0.05. The mean and standard deviation were used to describe the continuously measured variables, and the frequency and percentages were used to describe the categorically measured variables. The Kolmogorov-Smirnov statistical normality test and the histograms were used to assess the statistical normality assumption for the measured parameters. Parallel analysis was applied to assess the number of extractable factors from the COVID-19 worries and coping regulation questionnaires as well as the children's negative behaviors questionnaire. Categorical principal components analysis was used to compute a poverty index from parents measured socioeconomic factors (education, employment, residence, and family size). The non-parametric Friedman's test was applied to assess the statistical significance of the people's repeated measured perceptions, and the Wilcoxon's signed-rank non-parametric test was applied to compare the overall SEND-related coping strategies.

## Results

### Questionnaire reliability

The Cronbach's alpha test of internal consistency showed that the parental worries questionnaire was measured reliably (Supplementary Table A1).

### Participants' characteristics

One thousand eight hundred and forty-eight people have completed the online survey. The resulting descriptive analysis of the respondents' sociodemographic characteristics is shown in Table 1.

**Table 1:** Parent and child sociodemographic characteristics, *N* = 1,848.

	**Frequency**	**Percentage**
**Sex of the respondent**		
Female	595	32.2
Male	1,253	67.8
Age (years), mean (SD)		41.29 (8.18)
**Age group**		
20-−30 years	147	8
31-−40 years	784	42.4
41-−50 years	690	37.3
51-−60 years	227	12.3
**Relation to the child**		
Father	1,203	65.1
Mother	543	29.4
Others	102	5.5
**Educational level**		
No formal education	45	2.4
High school	621	33.6
Vocational degree/diploma	54	2.9
University degree	859	46.5
University degree—higher studies	128	6.9
Other	141	7.6
**Employment status**		
Full time paid job	1,119	60.6
Part time paid job	86	4.7
Volunteer job	6	0.3
Prime homemaker	322	17.4
Unemployed	92	5
Student	18	1
Retired	161	8.7
Other	44	2.4
**Do you (parent) have an anxiety disorder?**		
No	1,185	64.1
Yes	502	27.2
Prefer not to answer	161	8.7
Special need child's age (years), mean (SD)		9.66 (4.31)
**SN child age group**		
1–2 years	71	3.8
3–6 years	384	20.8
7–10 years	647	35
≥11 years	746	40.4
**SN child sex**		
Female	667	36.1
Male	1,181	63.9
**Region**		
Middle region	447	24.2
Eastern Provinces	325	17.6
Western Provinces	437	23.6
Northern Provinces	110	6
Southern Provinces	198	10.7
Other/missing	331	17.9

### The EFA of the coping regulation indicators of children with SEND

Exploratory factor analysis and principal axis factor analysis (PA) tests were applied to the correlations matrix between the perceived coping strategies of children with SEND, as rated by their parents. The Kaiser-Meyer-Olkin (KMO) index of sampling adequacy was satisfactory [=0.948, and Bartlett's test of sphericity indicated the absence of unwanted collinearity χ2_(91)_ = 45,287, *p* < 0.001] with a determinant index of = 0.005, suggesting the relevance of factor analysis for these measured indicators of coping regulation measured on children with SEND. As can be seen in Supplementary Table A2, the positive coping indicators (ventilation, seeking information, avoidance of stressful information, focusing and distraction, engagement in humor, rumination, and remaining positivistic, had loaded significantly and saliently (well ≥0.470) to the first factor namely the adaptive coping, and the remainder of the indicators measuring negative coping methods (self-isolation, aggressiveness, insistive and repetitive activity and speech, establishing routine, and parental avoidance) had loaded significantly and saliently (with loadings ≥ 0.532) to the second factor namely the maladaptive coping factor.

### EFA for worries and negative behaviors scales

The measured indicators of the negative behaviors of children with SEND had loaded significantly and positively to one latent factor that we considered as a single factor that may characterize children's ritualistic behaviors (RBs) before, during, and after the pandemic. This single factor explained a total of 58% of the shared variance between children's measured aspects of negative and ritualistic behaviors. The sampling adequacy and collinearity statistics showed that the factor analysis was tenable for these indicators of worry and negative behavior among parents and children, respectively, and all the negative behavior indicators had loaded saliently and significantly (loadings ≥0.480) to the single latent factor obtained from the exploratory analysis (Supplementary Table A2).

### Children's health, psychological wellbeing characteristics, and outcomes

Table 2 displays the yielded descriptive analysis of the health and medical condition as well the psychological wellbeing characteristics outcomes of children with SEND.

**Table 2:** Descriptive analysis of the health condition and medical and psychological history of children with SEND.

	**Frequency**	**Percentage**
**Does your child have intellectual disabilities?**		
No	666	36
Yes, mild-moderate	821	44.4
Yes, severe	361	19.5
**Is your child able to communicate their fear/anxieties?**		
No, not at all	733	39.7
Yes, non-verbal	316	17.1
Yes, both verbally & non-verbal	799	43.2
**Is your child taking any medications for psychiatric or psychological problems?**		
**(e.g., antidepressants, anti-anxiety medications, Ritalin, Prozac, Risperdal, etc.)**		
No	1,418	76.7
Yes	430	23.3
**Does your child have an anxiety disorder?**		
No	1,357	73.4
Yes	491	26.6
**SN child comorbidity**		
No	548	29.7
Yes	1,300	70.3
**Is your child aware of the existence of COVID-19?**		
No	1,193	64.6
Yes	655	35.4
How healthy would you say your child is? mean (SD)		3.88 (1.13)
Very Poor health	78	4.2
Poor health	133	7.2
Fair health	437	23.6
Good health	492	26.6
Very good health	708	38.3
How severe do you judge the medical issues of your child? mean (SD)		2.31 (1.20)
Not severe at all	610	33
Mild	446	24.1
Moderate	508	27.5
Severe	174	9.4
Very severe	110	6

**Figure 1:**
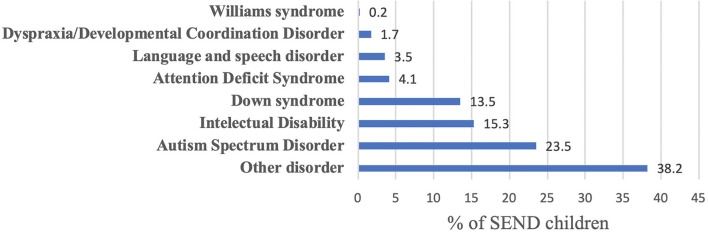
The SEND children's primary diagnoses.

The research participants were asked to indicate the primary diagnoses of their children with SEND, as can be seen in Figure 1.

**Figure 2:**
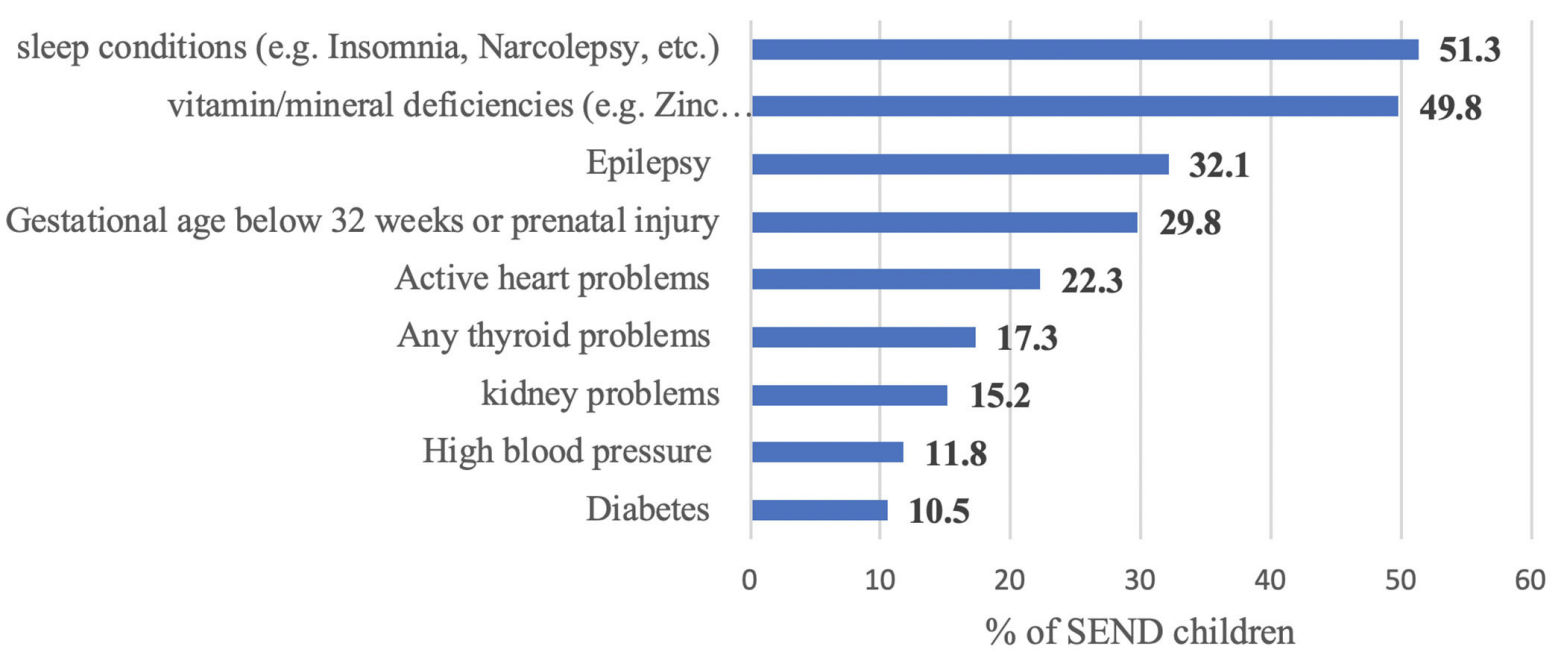
The SN childrens comorbidity types.

Parents were asked to indicate SEND comorbidity (Figure 2). Sleep problems, vitamin/mineral deficiency, and epilepsy were the top three reported comorbidities.

### Timeline of awareness about the pandemic and social distancing measures

Table 3 suggested that most of the participants had heard of the term, social distancing (SD) during or before May 2020. The self-rated mean concern of the participants about their family's safety upon hearing of social distancing was rated 2.98/5 points, indicating mild to moderate concerns. Only 52.1% advised that they implemented self-isolation and family social distancing upon governmental instructions.

**Table 3:** Descriptive analysis of people's perception of the COVID-19 pandemic and restrictions timeline.

	**Frequency**	**Percentage**
How concerned did you feel at the time for your family's safety when you first heard of COVID-19, mean (SD)		3.18 (1.33)
Not concerned at all	270	14.6
Mildly concerned	268	14.5
Moderately concerned	582	31.5
Highly concerned	311	16.8
Very concerned	417	22.6
How concerned did you feel at the time for your family's safety at the time you heard of social distancing? mean (SD)		2.98 (1.30)
Not concerned at all	279	15.1
Mildly concerned	373	20.2
Moderately concerned	604	32.7
Highly concerned	293	15.9
Very concerned	299	16.2
**Did your government suggest you should self-isolate?**		
No	283	15.3
Yes	1,565	84.7
How concerned (1–5) did you feel at the time for your family's safety?		2.77 (1.29)
Not concerned at all	193	20.5
Mildly concerned	209	22.2
Moderately concerned	283	30.1
Highly concerned	130	13.8
Very concerned	126	13.4
**Did the school/institution/place of employment that your child is attending**		
**close?**		
No	906	49
No, but we took our child out ourselves; date	256	13.9
Yes: Which date? ______	686	37.1
**If schools did not close, where is the child now**		
1 = “Still in school/institution/working”	20	2.3
Child is at home	852	97.7
Has your child with SN been infected with/or suspected they were infected with COVID- 19?	
No	1,800	97.4
Yes	48	2.6

### Parents' worries and anxiety before, at the start, and during the COVID-19 pandemic

Table 4 displays the descriptive yielded analysis and non-parametric Friedman's test analysis of the worries and anxiety of parents of children with SEND before, at the start, and during the COVID-19 pandemic. All parental perceived indicators of worry from the COVID-19 pandemic, have shown a significant rise across time, *p* < 0.050.

**Table 4:** Descriptive analysis of the worries and anxiety of parents of children with SEND throughout COVID-19 pandemic and restrictions timeline.

	**Mean (SD)-perception**				
	**Before**	**At start**	**Now**	***z*-statistic**	*p*-value^*^
**Parental perceptions about COVID**					
1. How anxious were/are you?	2.43 (1.33)	2.77 (1.43)	3.04 (1.38)	271.8	<0.001
2. How concerned were/are you about illness in general?	2.9 (1.44)	2.83 (1.42)	3.14 (1.37)	73.4	<0.001
3. How concerned were/are you about COVID-19?	2.63 (1.46)	2.90 (1.39)	3.20 (1.40)	231.6	<0.001
5. How concerned were/are you about your child's health?	2.96 (1.53)	3.17 (1.47)	3.38 (1.47)	213.1	<0.001
10. How concerned were/are you about the possibility that you will get ill?	2.73 (1.55)	3.03 (1.48)	3.20 (1.49)	236.9	<0.001
11. How concerned were/are you about the possibility that your child will get ill?	2.97 (1.64)	3.23 (1.55)	3.42 (1.53)	211.9	<0.001
4. How concerned were/are you about your child's safety with respect to COVID-19?	3.07 (1.6)	3.32 (1.51)	3.48 (1.49)	138.5	<0.001
13. How concerned were/are you about family conflict (fights, aggression)?	2.19 (1.50)	2.30 (1.49)	2.36 (1.53)	74.6	<0.001
14. How concerned were/are you about your financial/economic situation?	2.64 (1.59)	2.92 (1.54)	3.18 (1.58)	393.7	<0.001
9. How concerned were/are you about your child becoming bored?	2.97 (1.53)	3.22 (1.45)	3.32 (1.48)	153.3	<0.001
6. How concerned were/are you about the fact that your child has fewer occasions for social contact and interaction?	3.09 (1.56)	3.20 (1.49)	3.33 (1.50)	80.4	<0.001
7. How concerned were/are you that your child is not able to approach others?	2.68 (1.47)	2.96 (1.44)	3.15 (1.47)	215.02	<0.001
8. How concerned were/are you about your child's ability to cope with changes in his/her routine?	2.88 (1.50)	3.06 (1.44)	3.22 (1.47)	146.8	<0.001
12. How concerned were/are you about the loss of institutional (e.g., school, workplace) support for your child including interventions (language therapist, psychologist…)?	3.02 (1.64)	3.23 (1.56)	3.40 (1.59)	192.5	<0.001
15. How concerned were/are you about your ability to keep your child with SN entertained and motivated?	2.66 (1.5)	2.96 (1.46)	3.18 (1.51)	310.1	<0.001
16. How concerned were/are you about work/childcare balance related to looking after a child with SN?	2.66 (1.53)	2.84 (1.51)	3.03 (1.56)	217.9	<0.001

* 2-Sided *p*-value.

### Ritualistic behaviors of children with SEND

The resulting findings from the analysis of the negative behavior indicators of the children with SEND, shown in the bottom half of Table 5 suggested that the negative ritualistic behavior indicators of children with SEND have risen significantly in general from before the pandemic to the time when the pandemic has started then to during the pandemic, *p* < 0.001 each, respectively. Also, the negative behaviors of the children measured in the three time points showed that the children's insistence on things at home remaining the same was significantly higher during the pandemic compared to before the pandemic, *p* < 0.001. As well, the children's insistence on keeping their daily routines similar at the three time points showed that these children's insistence on similar daily routines during the pandemic was significantly higher, *p* = 0.020.

**Table 5:** Descriptive analysis of the worries and negative behaviors of children with SEND child throughout COVID-19 pandemic and restrictions timeline.

	**Mean (SD)-perception**				
	**Before**	**At start**	**Now**	***z*-statistic**	*p*-value^*^
**Special needs children's perceptions about COVID**					
1. How anxious Is/was Your child?	1.8 (1.28)	2.03 (1.40)	2.25 (1.49)	286.9	<0.001
2. How concerned Is/was Your child about illness in general?	1.73 (1.28)	1.86 (1.33)	1.99 (1.42)	150.6	<0.001
3. How concerned Is/was Your child about COVID-19?	1.71 (1.28)	1.87 (1.34)	1.98 (1.41)	160.1	<0.001
5. How concerned Is/was Your child about his own health?	1.76 (1.30)	1.90 (1.36)	1.98 (1.42)	145.6	<0.001
10 How concerned was/is your child about the possibility that they will get ill?	1.72 (1.28)	1.84 (1.33)	1.92 (1.39)	115.9	<0.001
11 How concerned was/is your child about the possibility that others will get ill?	1.71 (1.26)	1.83 (1.32)	1.90 (1.37)	99.4	<0.001
4. How concerned Is/was Your child about Your Family safety with respect to COVID-19?	1.70 (1.30)	1.84 (1.33)	1.93 (1.40)	157.7	<0.001
13. How concerned was/is your child about family conflict?	1.75 (1.33)	1.80 (1.32)	1.82 (1.34)	25.3	<0.001
14. How concerned Is/was Your child about his financial/economic situation?	1.70 (1.31)	1.77 (1.35)	1.85 (1.43)	95.3	<0.001
8 How concerned was/is your child about his or her loss of routine?	1.99 (1.40)	2.27 (1.45)	2.40 (1.52)	299.9	<0.001
12 How concerned was/is your child about the loss of institutional (e.g., school, workplace) support for your child including interventions (language therapist, psychologist…)?	1.88 (1.41)	2.04 (1.45)	2.12 (1.52)	139.7	<0.001
6 How concerned was/is your child about not being able to meet peers and friends?	1.96 (1.41)	2.13 (1.45)	2.25 (1.51)	187.8	<0.001
7 How concerned was/is your child about not being able to approach others?	1.87 (1.36)	2.04 (1.41)	2.17 (1.50)	189.5	<0.001
9 How concerned was/is your child about boredom?	2.10 (1.47)	2.29 (1.50)	2.43 (1.57)	224.1	<0.001
**Special needs children's behaviors about COVID19 pandemic**					
1. Insist on things at home remaining the same? (e.g., furniture staying in the same place, things being kept in certain places, or arranged in certain ways?)	1.97 (1.37)	2.03 (1.38)	2.10 (1.43)	72.75	<0.001
2. Get upset about minor changes to environment? (e.g., flecks of dirt on your clothes, minor scratches on your items?)	2.17 (1.42)	2.21 (1.42)	2.24 (1.44)	23.6	<0.001
3. Insist that aspects of daily routine must remain the same?	2.20 (1.45)	2.28 (1.45)	2.31 (1.48)	50.5	<0.001
4. Insist on doing things in a certain way or re-doing things until they are “just right”?	2.21 (1.43)	2.28 (1.42)	2.33 (1.47)	60.5	<0.001
5. Insist on wearing the same clothes or refuse to wear new clothes?	1.88 (1.34)	1.91 (1.33)	1.97 (1.39)	33.8	<0.001
6. Insist on eating the same foods, or a very small range of foods, at every meal?	2.30 (1.50)	2.31 (1.49)	2.34 (1.51)	25.1	<0.001

* 2-Sided *p*-value.

### Concerns about the pandemic of children with SEND

At these three time points, the general anxiety scores of the children with SEND showed that their anxiety scores when the pandemic started exceeded than that of those before the pandemic, *p* < 0.001. Also, their anxiety score during the pandemic time significantly exceeded their anxiety score before the pandemic, *p* < 0.001. Likewise, their anxiety score during the pandemic exceeded their general anxiety when it started, *p* < 0.001.

### Coping strategies and efficacy indicators of SEND during the pandemic time

#### Used coping methods

The analysis showed that the most used stress-relieving coping strategies of children with SEND during the pandemic time were: shielding behavior against the pandemic and associated stressful events, establishing daily routine and activities for themselves to relieve their stress, engaging in repetitive behaviors, and focusing their thoughts on positive aspects to view situations in a more positive manner.

#### Efficacy coping

According to the parents of children with SEND, the top efficacious coping methods used by their children, during the pandemic, were: distraction by establishing a daily activity routine to alleviate stress, shielding self against the stresses of the pandemic and its associated events, engaging in repetitive activities, focusing on positive aspects, and avoidance of negative thoughts and ruminating.

Table 6 displays the demonstrated coping strategies and efficacy indicators of children with SEND during the pandemic time.

**Table 6:** Descriptive analysis of the coping strategies and efficacy of the children with SEND during the COVID-19 pandemic and restrictions timeline.

	**Mean (SD)-perception**	
	**Use of coping methods**	**Efficacy**
1. In order to feel less stressed, my child avoids any information about it.	1.80 (1.32)	1.95 (1.40)
2. In order to feel less stressed, my child gets as much information as possible.	1.86 (1.31)	2.01 (1.42)
3. In order to feel less stressed, my child talks about it as often as possible	1.81 (1.28)	1.97 (1.38)
4. In order to feel less stressed, my child distracts him or herself as much as possible.	1.88 (1.30)	2.00 (1.38)
5. In order to feel less stressed, my child changes the way he or she is thinking about the situation	1.86 (1.28)	1.97 (1.35)
6. In order to feel less stressed, my child focuses on positive aspects/ views the situation in a different light (e.g., to have now more family time together)	2.01 (1.4)	2.10 (1.44)
7. In order to feel less stressed, my child tells jokes and engages in humor.	1.95 (1.37)	2.05 (1.43)
8. In order to feel less stressed, my child does not express negative emotions (i.e., suppression of emotions)	1.98 (1.34)	2.06 (1.39)
9. In order to feel less stressed, my child ruminates (i.e., thinks deeply about something)	1.93 (1.29)	2.03 (1.37)
10. In order to feel less stressed, my child engages in aggressive behaviors toward others around him/her	1.93 (1.36)	2.02 (1.43)
11. In order to feel less stressed, my child isolates himself/herself in his/her room, or another room of the house	1.86 (1.32)	1.94 (1.38)
12. In order to feel less stressed, my child engages in repetitive behaviors (asking the same questions repetitively, repeatedly washing their hands, rocking or other stereotypic behaviors (stimming), etc.).	2.03 (1.41)	2.12 (1.44)
13. I try to shield my child from the situation as much as possible	2.11 (1.47)	2.17 (1.52)
14. I try or my child tries to establish a routine in his/her daily life to lower the experienced stress FREQ	2.08 (1.41)	2.18 (1.42)

Table 7 displays the descriptive analysis of the children with SEND and their parents' overall perception scores yielded from the questionnaire. The overall parental pandemic worries score had risen significantly from before to the start than during the pandemic, *p* < 0.001, and overall worries between the three time points showed that parents' worries at the start of the pandemic exceeded significantly than their overall worries prior to the pandemic, *p* < 0.001.

**Table 7:** Descriptive analysis of the parents and overall perceived worries, anxiety, behavior, and coping strategies of their children with special needs before, at the start, and during the pandemic.

	**Mean**	**SD**
**Parental worries**		
Before COVID19	2.80	1.11
When COVID19 started	3.01	1.07
During pandemic	3.20	1.09
*Z*-statistic, *p*-value	288.2, *p*-value < 0.001	
**Parental general anxiety**		
Before COVID19	2.43	1.33
When COVID19 started	2.77	1.43
During pandemic	3.04	1.38
*Z*-statistic, *p*-value	271.8, *p* < 0.001	
**Special needs child worries**		
Before COVID19	1.81	1.08
When COVID19 started	1.96	1.13
During pandemic	2.06	1.18
*Z*-statistic, *p*-value	368.3, *p*-value < 0.001	
**Special needs children's ritualistic behavior**		
Before COVID19	2.12	1.08
When COVID19 started	2.17	1.08
During pandemic	2.22	1.11
*Z*-statistic, *p*-value	91.70, *p*-value < 0.001	
**Special needs children's anxiety**		
Before COVID19	1.80	1.28
When COVID19 started	2.03	1.39
During pandemic	2.25	1.49
*Z*-statistic, *p*-value	286.9, *p* < 0.001	
**Special needs child coping regulation**		
Adaptive coping	1.85	1.04
Maladaptive coping	1.99	1.08
Efficacy coping	2.04	1.04
*Z*-statistic, *p*-value	7.914, *p* < 0.001	

The overall worries of the children with SEND from the pandemic, as can be seen in Table 6 show that their overall worries from the pandemic differed significantly between the three measured time points, *p* < 0.001, and negative RB score had risen significantly across the pandemic timeline. RB score during the pandemic had exceeded than that prior to the pandemic and that at the start of the pandemic, *p*-value < 0.017 and *p*-value < 0.001, respectively. Additionally, the maladaptive coping score of children with SEND children had exceeded their adaptive coping score significantly, *z* = 7.914, *p*-value < 0.001. The children's efficacy coping score was measured with 2.04/5 points according to their parents' rating.

Table 8 displays the bivariate correlations between the children with SEND and their parent-measured perceptions. The negative RB of the children with SEND during the start of the pandemic had correlated significantly positively with their negative behavior during the pandemic, *r* = 0.951, *p* < 0.010. The children's general worries at the start of the pandemic had correlated significantly and positively with their anxiety, adaptive, maladaptive, and coping efficacies as well as with parental anxiety scores, *p* < 0.010 each, respectively. The general anxiety score of the children with SEND had also correlated significantly positively with their adaptive, maladaptive, and efficacy coping scores as well as with their parental worries and anxiety scores. Not only so, but also the adaptive coping efficacy of the children with SEND had converged significantly and positively on their maladaptive coping scores and on their coping efficacy scores, *p* < 0.001, denoting that these children may have been using both adaptive and maladaptive coping methods dually.

**Table 8:** Bivariate correlations between the measured outcomes of the children with SEND and Pearson's correlation test.

	**Behavior-WS**	**Behavior now**	**Worry before**	**Worry at start**	**Anxiety before**	**Adaptive cope**	**Maladaptive cope**	**Efficacy**	**Parent anxiety before**	**Parent anxiety at start**
Children's negative behavior when COVID started	1									
Special needs child Behavior Now	0.951^**^	1								
Special needs child worries before COVID	0.408^**^	0.396^**^	1							
Special needs child worries when COVID started	0.420^**^	0.405^**^	0.906^**^	1						
SEND anxiety before COVID	0.315^**^	0.294^**^	0.689^**^	0.618^**^	1					
Adaptive coping Special Need child	0.322^**^	0.319^**^	0.458^**^	0.491^**^	0.308^**^	1				
Maladaptive coping Special Need child	0.354^**^	0.364^**^	0.366^**^	0.401^**^	0.247^**^	0.722^**^	1			
Efficacy coping SEND child	0.327^**^	0.338^**^	0.414^**^	0.459^**^	0.273^**^	0.867^**^	0.805^**^	1		
Parent anxiety before COVID	0.217^**^	0.228^**^	0.273^**^	0.226^**^	0.262^**^	0.116^**^	0.109^**^	0.113^**^	1	
Parent anxiety when COVID started	0.165^**^	0.157^**^	0.189^**^	0.232^**^	0.132^**^	0.083^**^	0.100^**^	0.085^**^	0.441^**^	1

** Correlation is significant at the 0.01 level (2-tailed).

## Discussion

This is the first national study addressing SEND and their caregivers' perspectives during the first wave of the COVID-19 pandemic in Saudi Arabia. Our study addressed the health and medical condition, psychological wellbeing characteristics, and outcomes including the coping behaviors of children with SEND. Similarly, we reported SEND's caregivers' perspective and their worry levels across different time points during the pandemic. Nonetheless, we also highlighted the coping behaviors of children with SEND and their relation to the anxiety levels of their caregivers.

In terms of sample characteristics, we found that 44.4% of the children with SEND had mild intellectual disabilities, and 19.5% had severe disabilities, a notion reported before, that the severity of intellectual disability tends to be mild rather than severe (24). Furthermore, our results indicate that 23.3% of children with SEND receive psychiatric medications; however, the indication was not reported. In a study assessing the needs of children with special health care needs (CSHCN), approximately half of CSHCN were identified as having special needs due to the needed additional medication including psychotropics (25). However, when the parents were asked to rate the general health (GH) of their children with SEND, the collective GH was rated from fair to good health in general, while 11.4% of the children were considered to have poor to very poor health. A similar study reported that although the CSHCN had poorer health status than children without special needs, many CSHCNs were reported to be in good health. This suggests a broad spectrum of severity of illnesses within the CSHCN group (26). Our results also indicated significant comorbidities among children with SEND (70.3% with another comorbidity), with 51.3% of them having sleep problems. Our finding concerning sleep is not surprising, as sleep problems are very relevant in diagnoses associated with special needs, such as NDDs (in which up to 80% of children with NDDs might have sleep difficulties) (27).

Our results also described the primary diagnosis of children with SEND, revealing that most children had one of those three diagnoses: autism spectrum disorder, down syndrome, and intellectual disability. In a study assessing CSHCN, the results indicated that the prevalence of CSHCN was 12.2%, and 91.8% of them had the three domains of health care needs, with the most prevalent conditions requiring special health care were sensory and cognitive impairments and impaired mobility (28). We believe that our findings might be colored by the nature of the Ministry of Social Affairs system in registering beneficiaries and the fact that the Autism Center of Excellence had contributed a lot to the data collection. One aspect we attempted to address in our study was the ability of the children with SEND to communicate their fear or anxieties, which indicated several means of communication. Our finding is consistent with the general notion that children with SEND, e.g., with intellectual disabilities, may struggle in terms of communicating and may need support with understanding and expressing themselves (29).

Simultaneous with the government's effort to raise awareness regarding the novel coronavirus pandemic, the majority of the participants in this study have been informed about the pandemic by March 2020 (30). According to the available data, families of children with SEND and the children themselves endure more significant repercussions of the pandemic including mental health concerns (31, 32). However, in this study, parents of children with SEND showed moderate concern regarding the situation which is also documented in other available studies (33). On the other hand, some reports indicated no difference in the level of psychological stress perceived across families having children with SEND prior, in the midst, and after COVID-19 measures (34).

In our study, we found that parents of children with SEND developed more concern as governmental COVID-19 mandates including social distancing and isolation were implemented. Overall worries about their own safety and that of the family are indicated to be moderate, a finding confirmed in former studies (35). Nevertheless, the implication of isolation and social distancing has resulted in a huge psychological strain on parents. According to reports inspecting the reason for parents' mental ill health, is their having to accommodate the demands of their children with SEND due to special need services and school closure (32, 35). Nearly 49% of the participants confirmed ongoing school and work placements for their children with SEND; however, they chose to keep them at home; this is linear with other studies that related this decision to the vulnerability of this population (31).

The worries of the parents of the children with SEND showed a significant rise across time, from before the pandemic, to the start of the pandemic, until during the pandemic, which supports previous studies conducted among parents of children with SEND living in China (32) and in UK (31). However, parents' concerns over their children's illness showed no significant change between before the pandemic and the time it had started, which comes in line with the findings of the study in China in which parents were not concerned about the impact of the pandemic on the health of their children (32). It was proposed that it is due to less severe COVID-19 manifestations on children (36). Similarly, parents' concerns over their own illness and their children losing the social interaction showed no difference between the time points, before and the start of the pandemic, in our results which contradicts the results of the study done in China (32). Regarding family-related worries, parental concerns over getting family conflicts did not increase in our study, which was consistent with the study in China (32). A possible reason for this finding is the increased family bonding which is a way of coping with the stress of the pandemic, as a positive change in the familial bond was observed during the lockdown in Saudi Arabia (37). Importantly, the parental perceived general anxiety had risen significantly when the pandemic started, which agrees with the study done in Saudi Arabia among parents of children with autism spectrum disorder (ASD) (38) and in China (32) and UK (31) among parents of children with SEND.

Concerning the general anxiety and worries of children with SEND, a significant increase has been shown across different time points, before, at the start of, and during the pandemic, which is consistent with the study done in the UK (31), that showed a similar rise in a wide range of worries, including worries over COVID illness, health, social-related worries involving friends and approaching others, and worries about loss of institutional support, and getting bored. In our study, the concerns of children with SEND about the other people around them getting sick did not show any significant difference among the various times in relation to the COVID outbreak, while those same concerns decreased over time among children with SEND, in the study by Sideropoulos et al. (31). The former finding can be attributed to difficulties of children with disabilities, especially intellectual disabilities, and ASD, to understand the situation of the pandemic or communicate their worries (18). The latter can be explained by the development of adaptive coping with time, which could have improved the worries. Additionally, the concerns about their family conflicts and family finances did not change over time between before and the start of the pandemic according to the children with SEND, which agrees with the study among Chinese children with SEND (32). One explanation is that children are unlikely to have financial responsibility. This contradicts the findings in UK where children with SEND showed increasing worries about family finances (31). The discrepancy in findings could be attributed to cultural differences. In terms of the negative RB of children with SEND, a significant increase was found across the three time points (before, start, and during the pandemic), which is consistent with a study by Amorim et al., among children with ASD (39). This contrasts with a study conducted in Italy that showed no worsening of repetitive behavior. However, their findings were probably attributed to the region, from where the sample was taken, being one of the least COVID-19 affected regions in Italy (40).

In terms of coping strategies, our results show that parents tried to shield their children from SEND and establish daily routines and activities commonly to cope with the stress of the pandemic, and both strategies were cited to be the most effective. Similarly, many individuals with SEND have been shielded due to the increased risk of infections and severe forms of COVID-19 (41). As a result, these individuals' access to the support provided by schools and other help and rehabilitation services has been largely restricted, leading to increased depression and anxiety and worsening of problematic behaviors (42, 43). One of the important factors explaining this effect is the loss of daily routine and disruptions to regularly scheduled activities (44, 45). Indeed, such changes in daily routine and habits present a challenge to children with SEND, who rely on a clear structure and routine for daily functioning (46). In our study, the establishment of daily routines was among the most frequently used and effective strategies to cope with the pandemic stress for children with SEND. In line with our findings, Jacques et al. reported that the establishment of daily routines was a key facilitating factor for coping among 67% of parents of children with ASD (47). Multiple studies also highlighted the need for maintaining regular routines and structured activities during the pandemic (48, 49).

When looking at the negative RBs, our findings indicate that their presence during the pandemic was related to their presence during the pandemic, with the general worries of the children at the beginning and during the pandemic, and to their anxiety before and after the pandemic. Similarly, a study in the UK reported an increase in restricted and repetitive behaviors when the pandemic started (50). Such an association can be explained by the disruption of routines and the loss of helping services provided by schools and other rehabilitation services due to the lockdown (51). Another explanation is the increase in general worries and anxiety among children with SEND during the pandemic, as previous research suggests that repetitive behaviors are associated with varying degrees of anxiety among people with ASD (52). Our results suggest that adaptive and maladaptive coping behaviors increased among children with SEND during the pandemic. In line with our results, a study by Tokatly Latzer et al. reported an increase in negative and positive behaviors among children with ASD during the COVID-19 lockdown (53).

In addition to the findings from other studies, the current study observes that parental anxiety is associated with anxiety and worries among children with SEND. Studies both before (54) and during (31, 55) the pandemic reported similar links between the caregiver's anxiety and severity of anxiety among children with SEND. Additionally, our findings suggest that parental anxiety prior to the pandemic was associated with their anxiety at the beginning and during the pandemic. Furthermore, negative RBs at the beginning were associated with parental anxiety before and at the beginning of the pandemic. Similarly, multiple studies reported that parental distress was associated with the worsening of symptoms in children with NDDs (56–58). Contrary to our findings, a longitudinal study from Italy found no differences in parental wellbeing and children's behavioral problems before and after the lockdown (34). These differences might be explained by the positive impact some parents reported as the positive outcomes of the lockdown experience, such as more family time together, better child–parent relationship and improvement in some symptoms of their children with SEND (40). Despite the paucity of published work on SEND population in Saudi Arabia, a recent report did try to assess parents' worries about their children with SEND getting infected and recommended special education facilities to establish policies that address their worries (59).

## Conclusion

Before and throughout COVID-19, children with SEND, and their caregivers had high anxiety levels. Over time, there had been a marked increase in the anxiety of children with SEND (from before the pandemic to when it had started to during the pandemic). The anxiety, adaptive, maladaptive, and coping efficacies, and parental anxiety scores of the children with SEND at the beginning of the pandemic were substantially and positively associated.

## Limitations and future directives

This extensive study examines the psychological effect of the pandemic on children with SEND and their parents in Saudi Arabia; however, limitations are attributed to the study design and infrastructure of the Saudi SEND service provision. A significant limitation of the study comes from the data collection technique; the data presented in this study were derived from a cross-sectional survey that was only available online. Hence, families with low socioeconomic status could have had limited access to technology to complete the survey, making it less likely for them to be represented in our data. Moreover, some parents may have been unable to complete the survey due to overwhelming care and work responsibilities. Additionally, parents with more than one child must use another device to fill out a second survey, as the system was designed to prevent duplication.

While several disability service providers and support groups with national coverage circulated the survey to parents within their networks, there is a risk of selection bias that impacts the generalizability of the survey results. The survey was sent to SEND caregivers *via* text messages to all beneficiaries of the Ministry of Human Resources and Social Affairs, the Autism Center of Excellence social media accounts, and the Authority for Persons with Disabilities. Surveys typically fail to include an adequate representation of rural and remote families. However, we regard it as a strength of our research that there were responses from all regions of the country with relatively similar response rates from the three major regions in Saudi Arabia.

Additionally, reliance on parent report questionnaires is an explicit limitation of this study, especially when it comes to older children who are verbal with adequate cognitive skills and whose experience could have been better captured through self-administered questionnaires or virtual assessments. Parents' reports on the abilities of their children could be influenced by reporting biases, such as the Horn effect, i.e., parents of children with greater symptom severity may have rated higher scores for adverse impact. At the same time, they may not be able to reflect on their children's experience of mild anxiety and depressive conditions. Hence, to generalize our considerations, we would have to assess for longitudinal outcomes, which can be represented through parental observation and formal assessments.

Additionally, the study is a part of an international project; it utilized secondary data from the international collaboration responses to the pandemic. Five main conditions were listed as options, leaving the sixth choice as “others” for parents to fill, which could have discouraged the caregivers of children/adolescents with other conditions from listing their diagnosis. Similarly, the survey centralized on assessing anxiety as an indication of overall mental health.

Finally, even though the age range was broad (from 1 to 18 years old), it was essential to capture, while interpreting with caution, since the level of care needed for a toddler can be disproportionate to that given to an adolescent; however, not always.

## Recommendation

Children with SEND are a vulnerable part of the community with their own specific mental and general health needs. We recommend, developing a systematic and proactive tool to support SEND and their caregivers at the community level during pandemics and national health crises with more emphasis on mental health needs.

## Data availability statement

The raw data supporting the conclusions of this article will be made available by the authors, without undue reservation.

## Ethics statement

The studies involving human participants were reviewed and approved by the Institutional Review Board at King Saud University (approval #20/0065/IRB). The patients/participants provided their written informed consent to participate in this study.

## Author contributions

SA, M-HT, ASA, AHA, AAlm, MSA, and FA conceptualized the study, analyzed the data, and wrote the manuscript. SA, M-HT, ASA, FB, SHA, MHA, RA, AAlk, and MA contributed to the study design, collected, analyzed, interpreted data, and edited the manuscript. All authors reviewed and approved the final version of the manuscript.

## Conflict of interest

The authors declare that the research was conducted in the absence of any commercial or financial relationships that could be construed as a potential conflict of interest.

## Publisher's note

All claims expressed in this article are solely those of the authors and do not necessarily represent those of their affiliated organizations, or those of the publisher, the editors and the reviewers. Any product that may be evaluated in this article, or claim that may be made by its manufacturer, is not guaranteed or endorsed by the publisher.
