# Supplementary material for: Mental health impact of COVID-19 on Saudi families and children with special educational needs and disabilities in Saudi Arabia: A national perspective
Source: Front Public Health. 2022 Sep 27;10:992658. doi: 10.3389/fpubh.2022.992658 (PMC9551570; doi:10.3389/fpubh.2022.992658)
Supplement: Supplementary file 1 [file Table_1.DOCX]

| **Table-A1: Reliability analysis of the measured questionnaires.** | | |
| --- | --- | --- |
|  | *Number of items* | *Cronbach's alpha* |
| Parent worries questionnaire | 15 | 0.94 |
| SEND child worries questionnaire | 14 | 0.96 |
| SEND children's behavioral scale | 6 | 0.87 |
| SEND children's used Coping methods scale | 14 | 0.93 |
| SEND children's Coping efficacy scale | 14 | 0.95 |

| **Table-A2 : Principal Components Analysis Promax Rotated Pattern Matrix factor solution for SEND children’s coping strategies scale** | | |
| --- | --- | --- |
|  | **Extracted Component** | |
|  | **Adaptive coping** | **Maladaptive coping** |
| In order to feel less stressed, my child talks about it as often as possible | .925 |  |
| In order to feel less stressed, my child gets as much information as possible | .901 |  |
| In order to feel less stressed, my child avoids any information about it | .843 |  |
| In order to feel less stressed, my child focuses on positive aspects/ views the situation in a different light (e.g. to have now more family time together) | .809 |  |
| In order to feel less stressed, my child changes the way he or she is thinking about the situation | .794 |  |
| In order to feel less stressed, my child tells jokes and engages in humor | .772 |  |
| In order to feel less stressed, my child distracts him or herself as much as possible | .711 |  |
| In order to feel less stressed, my child ruminates (i.e., thinks deeply about something) | .515 |  |
| In order to feel less stressed, my child does not express negative emotions (i.e., suppression of emotions) | .470 |  |
| In order to feel less stressed, my child isolates himself/herself in his/her room, or another room of the house |  | .887 |
| In order to feel less stressed, my child engages in aggressive behaviors towards others around him/her |  | .859 |
| In order to feel less stressed, my child engages in repetitive behaviors (asking the same questions repetitively, repeatedly washing their hands, rocking or other stereotypic behaviors (stimming), etc.). |  | .803 |
| I try or my child tries to establish a routine in his/her daily life to lower the experienced stress |  | .580 |
| I try to shield my child from the situation as much as possible |  | .532 |
| Extraction Method: Principal Component Analysis.   Rotation Method: Promax with Kaiser Normalization. | | |
| a. Rotation converged in 3 iterations. | | |
